# In a Heartbeat: Light and Cardiovascular Physiology

**DOI:** 10.3389/fneur.2017.00541

**Published:** 2017-10-20

**Authors:** Sarah L. Chellappa, Ruta Lasauskaite, Christian Cajochen

**Affiliations:** ^1^Medical Chronobiology Program, Division of Sleep and Circadian Disorders, Brigham and Women’s Hospital, Boston, MA, United States; ^2^Division of Sleep Medicine, Harvard Medical School, Boston, MA, United States; ^3^Centre for Chronobiology, Psychiatric Hospital of the University of Basel, Transfaculty Research Platform Molecular and Cognitive Neurosciences, University of Basel, Basel, Switzerland

**Keywords:** light, non-image-forming system, circadian rhythms, cardiovascular physiology, heart rate variability

## Abstract

Light impinging on the retina fulfils a dual function: it serves for vision and it is required for proper entrainment of the endogenous circadian timing system to the 24-h day, thus influencing behaviors that promote health and optimal quality of life but are independent of image formation. The circadian pacemaker located in the suprachiasmatic nuclei modulates the cardiovascular system with an intrinsic ability to anticipate morning solar time and with a circadian nature of adverse cardiovascular events. Here, we infer that light exposure might affect cardiovascular function and provide evidence from existing research. Findings show a time-of-day dependent increase in relative sympathetic tone associated with bright light in the morning but not in the evening hours. Furthermore, dynamic light in the early morning hours can reduce the deleterious sleep-to-wake evoked transition on cardiac modulation. On the contrary, effects of numerous light parameters, such as illuminance level and wavelength of monochromatic light, on cardiac function are mixed. Therefore, in future research studies, light modalities, such as timing, duration, and its wavelength composition, should be taken in to account when testing the potential of light as a non-invasive countermeasure for adverse cardiovascular events.

## Out of Sight: Impact of Light on Behavioral Brain Responses

The mammalian eye senses light for fulfilling its dual function *via* two detecting systems: (1) the classical visual system that serves for image formation (1) and (2) the non-image-forming (NIF) system, which besides unconscious vision and activating early visual systems (2) affects a myriad of physiological and behavioral responses (3–7). Importantly, while these two systems differ in terms of their functions, growing evidence indicates that their complete dichotomy is outdated at the eye and brain levels (8–11). Thus, it is more likely that numerous outputs of our physiology and behavior are affected by a multi-dimensional system which can be divided into different networks. NIF effects are mostly—but not exclusively (10)—driven by melanopsin—a photopigment found in intrinsically photosensitive retinal ganglion cells (ipRGC) (3), which also play an important role as relay to transmit NIF effects coming from the classical visual system (9). These photoreceptors essentially detect environmental irradiance and exhibit maximal sensitivity to short wavelength light (blue, peak sensitivity ~480 nm) (3, 4, 12). Melanopsin ipRGCs typically display a low spatial resolution, long response latencies in contrast to fast responding cones and rods, can integrate photic energy even beyond the duration of a given light exposure, and are also involved in the detection of motion and, patterns (2–4, 9, 10, 13–15). These slow kinetics could be a potential factor underlying long-term light effects on human physiology and behavior, such that—apart from its acute effects (16)—it also impacts on, i.e., skin temperature, melatonin rhythms, and sleep–wake regulation, even after light exposure is over (17–20).

The ipRGCs directly project *via* the retino–hypothalamic tract to the suprachiasmatic nuclei (SCN) within the anterior hypothalamus, commonly deemed as the primary circadian oscillator (7, 21). The SCN, in turn, projects multisynaptically to the pineal gland (22) (associated with melatonin synthesis) and to numerous brain regions that receive input from the visual photoreceptor system, as, for instance, the lateral geniculate nucleus, superior colliculus, and olivary pretectal nucleus which is a essential node for the pathway for pupillary constriction (5, 23, 24). The melanopsin-expressing ipRGCs are also directly connected to regions which are regulating the sleep–wake cycle (25–27), including the ventrolateral preoptic nucleus (VLPO; linked to sleep–wake regulation), the subparaventricular nucleus/zone of the hypothalamus (SPVZ; linked to sleep regulation and to motor activity), and the lateral hypothalamus containing orexin (hypocretin) wake-promoting neurons (23, 24, 28).

**Figure 1:**
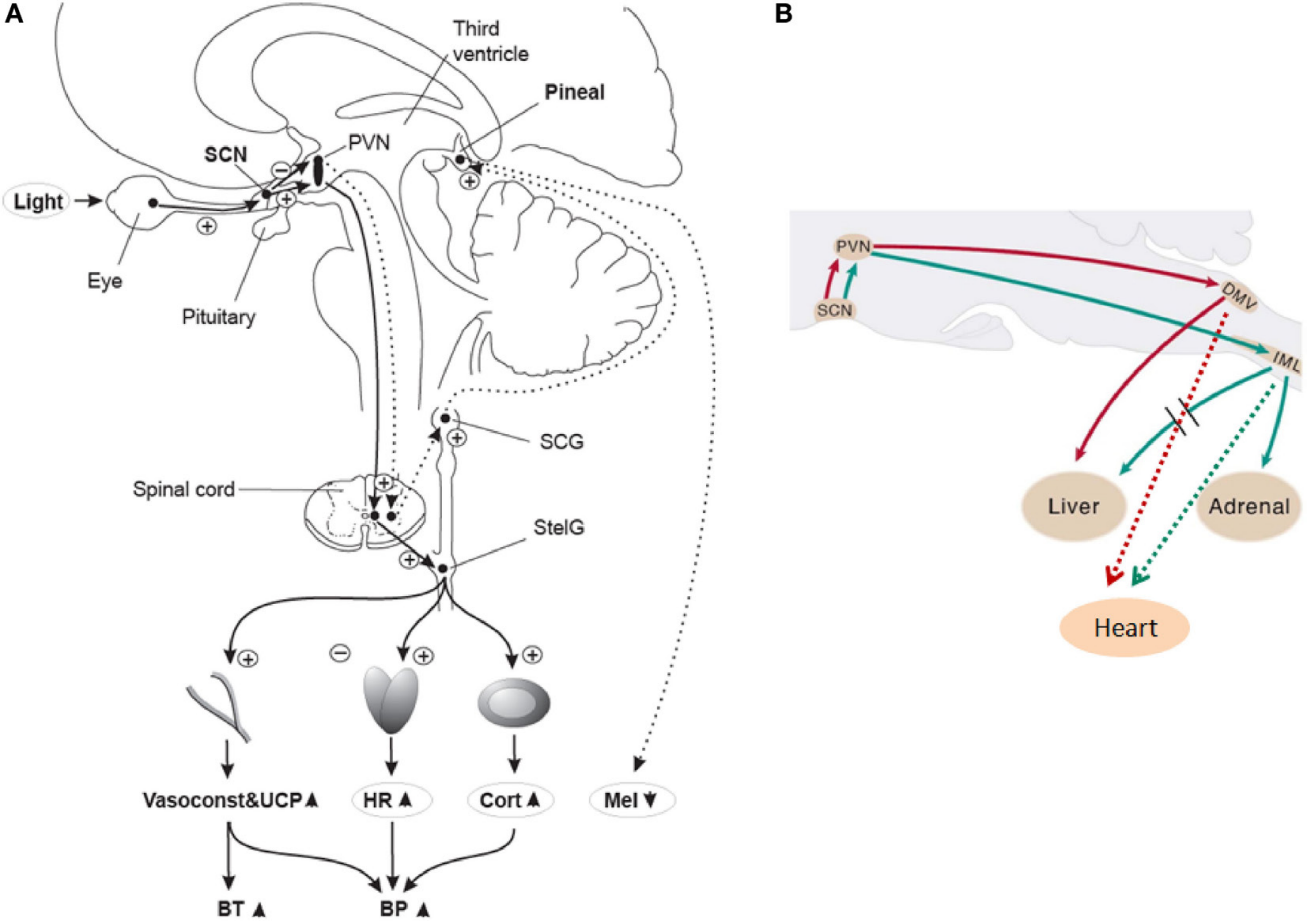
**(A)** Putative light mechanisms on human cardiovascular system *via* the suprachiasmatic nuclei (SCN). Human SCN potentially sends different projections to different parts of the PVN to inhibit melatonin in the pineal gland, while stimulating most other SCN-driven rhythms (e.g., HR and cortisol) after light exposure. BP, blood pressure; BT, body temperature; Cort, cortisol; HR, heart rate; Mel, melatonin; PVN, paraventricular hypothalamus nucleus; SCG, superior cervical ganglion; StelG, stellate ganglion; Vasoconstr & UCP, vasoconstriction and uncoupling protein. Continuous lines: active pathways, dotted lines: suppressed pathways. “Plus” signs: stimulation; “minus” signs: inhibition. Reproduced with permission from Scheer et al. (42). **(B)** Putative sympathetic and parasympathetic outputs from the SCN to peripheral organs *via* neurons of the rat brainstem. DMV, dorsal motor nucleus of the vagus; IML, intermediolateral spinal cord column. Red lines: parasympathetic output. Green lines: Sympathetic output. Dashed red and green lines correspond to *potential* output pathways. Modified from Kalsbeek et al. (34).

Early animal anatomical and functional studies showed that light information conveyed to the SCN affects melatonin secretion from the pineal gland (22, 29–31) and glucocorticoid secretion from the adrenal cortex (32, 33). Furthermore, in rodents, the SCN may influence parasympathetic output to the heart *via* connections with pre-autonomic neurons within the hypothalamus, which may enable the 24-h sympathetic-parasympathetic balance of autonomic cardiac inputs (34). Collectively, these data suggest that the SCN can transfer its time-of-day information to various organs throughout the body like the cardiovascular system (see Figure 1 for a putative schematic diagram on the anatomical mechanisms for light modulation on the cardiovascular system via the SCN). Interestingly, hypertension seems to be related to changes in both SCN morphology and function in rodents (35, 36) and humans (37–39). This potential disturbance in hypertension-related circadian regulation, together with the incidence of tachycardia prior to the onset of hypertension (40), correspond to some of the earlier findings that highlighted a putative role of the SCN as a mediator of cardiovascular physiology (41).

NIF effects are essential for the circadian entrainment to the solar 24-h light/dark cycle and can both change the amplitude and the phase of outputs of the circadian system, including, but not limited to hormone secretion (i.e., melatonin and cortisol), body temperature, sleep–wake cycle, and cardiovascular function in humans (17–20, 43–45). Although photoreceptors are not directly accessible in humans, two studies have provided a putative role of ipRGCs for light affecting cognitive brain responses, including frontal cortical areas associated with executive cognitive control (46, 47). Furthermore, in conjunction to light’s wavelength, properties like intensity, duration, and timing are essential in discovering how light differentially impacts on human physiology [for reviews, see Ref. (16, 48)]. For instance, light exposure at night elicits a suppression of melatonin levels, as well as a decrease in subjective and objective indicators of sleepiness (e.g., subjective sleepiness, theta activity in waking EEG, occurrence of slow eye movements) (19, 44, 49–51). Recently, it was shown that exposure to progressively dynamic morning light can directly influence sleep inertia, well-being, and cortisol levels in humans (52–54).

Despite growing evidence for a plethora of NIF effects of light on our physiology, relatively few studies have investigated how it impacts on cardiovascular control. In the next section, we address some of the evidence which speaks to how targeted light exposure—e.g., morning vs. night light exposure, different wavelengths, illuminance levels, dynamics—affects cardiovascular function.

## Light up the Heart: Impact of Light Exposure on Cardiovascular Control

The heart is the propelling “force for the delivery of oxygen and nutrients, for the disposal of waste and for the distribution of heat” (42). These cardiometabolic demands do not occur at an even rate throughout the 24-h day, but rather heavily change their dynamics over the day and night (55). Importantly, the heart contains a peripheral clock that markedly modulates cardiovascular physiology (e.g., gene and protein expression, extracellular stimuli/stresses responsiveness) and its daily rhythmicity (56). Importantly, maximum risk for cardiovascular incidents is in the morning hours between 06:00 a.m. and 12:00 a.m. (57–61). Some other evidence shows an increase in occurrences in the evening (between 06:00 p.m. and midnight), hinting to a possible bimodal pattern (62). This “morning shift” in key cardiovascular regulatory mechanisms is an important characteristic of ischemic diseases like brain vascular disease, cerebral infarction, and myocardial infarction (63). Furthermore, hypertensive patients show a compromised cardiovascular anticipation to the activity period that may increase the risk of cardiovascular incidents in the early morning hours (39, 42, 58, 64). Yet, these adverse cardiovascular events in the morning hours cannot be explained by solely daily rhythm in external factors like body position and activity (65). Instead, it is more likely that they might be associated with circadian changes in blood pressure, vascular tone, catecholamines, platelet aggregation, increase in plasminogen activator inhibitor-1, heart rate (HR), and variation in beat-to-beat interval (63, 66, 67). Clinical findings also suggest a small (but not trivial) 1.28-fold higher rate of acute myocardial infarction in a wide window (06:00 a.m. to 12:00 a.m.) in comparison to the rest of the day (57), bimodal peaks in the morning and evening hours (62), and stress-related contributors to adverse cardiovascular events (68). Thus, a dysfunction of the circadian clock may possibly be a risk factor for cardiovascular diseases, contributing, to some degree, to increased HR and heart rate variability (HRV) in the morning. Given that (1) the endogenous circadian timing system is best synchronized to the 24-h cycle by light (69) and (2) the SCN modulates the adrenal and the heart (33, 42), it is, therefore, reasonable to infer that exposure to different light modalities may affect cardiovascular function [e.g., HR, HRV, pre-ejection period (PEP)]. Thus, specific light properties, such as timing, exposure duration, intensity, wavelength, and dynamics, may well determine the magnitude of such effects.

Earlier human studies indicate that resting HR is affected by the day/night cycle and on the light level (41, 70). Accordingly, bright polychromatic light exposure (10 min of either no light, light at 100 lux or light at 800 lux) increased resting HR in the early morning hours, particularly during exposure to light at 800 lux (41), with no effects on the vagal tone, as indexed by the root mean square of the successive differences of the inter-beat interval (RMSSD) (70), which is a valid index of vagal tone (71). Furthermore, exposure to bright polychromatic light at 5,000 lux for 4 h during either the day (12:00 p.m. to 04:00 p.m.) or night (12:00 a.m. to 04:00 a.m.; thus also including the early morning hours prior to an individual’s wake-up time) suggest a time-of-day dependency: HR increased due to being exposed to bright light at night, but not during daytime (72). One plausible explanation for the time-of-day dependency of light on HR in the early morning hours might be related to the endogenous increase in sympathetic cardiac activity during this time window (42). The light-dependent increase in sympathetic tone may be due to light exposure in the morning hours, as sympathetic muscle nerve activity increases with morning light exposure (73). HR is also affected by light in a phase-dependent manner, which goes in concert with phase-dependent influence on sympathetic tone by morning, but not evening light (41). However, human sympathetic modulation estimated as pre-ejection period (PEP) is relatively uncoupled from the endogenous (i.e., regulated by the CNS) circadian drive and is mainly influenced by prior activation (74). Light can elicit acute cardiovascular physiological effects that depend on properties beyond timing and duration, such as its wavelength. Indeed, a 2-h exposure to monochromatic blue light (460 nm) in the late evening led to increased HR as compared to 550-nm monochromatic (green) light, indicating a key role for melanopsin NIF photoreceptors in modifying human HR (17).

Light effects on HRV show mixed-results in humans (75–77). For instance, higher illuminance (1,000 lux bright polychromatic white light) increased the low-frequency to high frequency (LF/HF) HR power ratio as compared to baseline (vs. 200 lux bright polychromatic white light), which may suggest a relative increase in cardiac sympathetic activity under higher illuminance (77). Furthermore, evening (~21:00 h) exposure to 10-min of red, green, and blue fluorescent lights of 700 lux, preceded and followed by 15-min of darkness, decreased the absolute HF HR power in the episode of darkness only following blue light episode (75), suggesting that HF HR is specifically sensitive to high frequency (blue) light. Conversely, exposure to 5-min of blue, red, and white fluorescent lights may lead to a decrease in absolute HF power following exposure to only red light (76). The dissimilarity of these earlier human findings may be associated with differences in body posture (78) and respiratory frequency, the later being affected by light depending on its color (79). A recent human study measured autonomic cardio-respiratory outputs (i.e., electrocardiogram and respiration) during 6-min exposure to colored OLED (red, green, and blue lights), which was preceded and followed by 3-min of darkness under paced breathing (15 breaths/min) (80). These cardio-respiratory measurements were repeated after 45 min with melanopsin-stimulating photon flux density (MSPFD) of 0.00, 0.10, and 0.20 µmol/m^2^/s, respectively. Accordingly, HF (0.20–0.30 Hz) power had a greater decrease with blue light in comparison to red and green lights. Furthermore, HF power decreased with blue light with 0.20 µmol/m^2^/s MSPFD, but not with that with 0.10 or 0.04 µmol/m^2^/s, suggesting a dose-dependent effect to blue light exposure. HF power especially between 0.15 and 0.40 Hz, also called the respiratory band, reflects vagal tone to control HR (80). In this context, one may speculate that blue OLED light exposure may result in a vagal cardiac suppression through melanopsin-dependent NIF effects, which might ultimately shift the state of our body from a resting mode to an arousal one similar to animal data (27).

**Figure 2:**
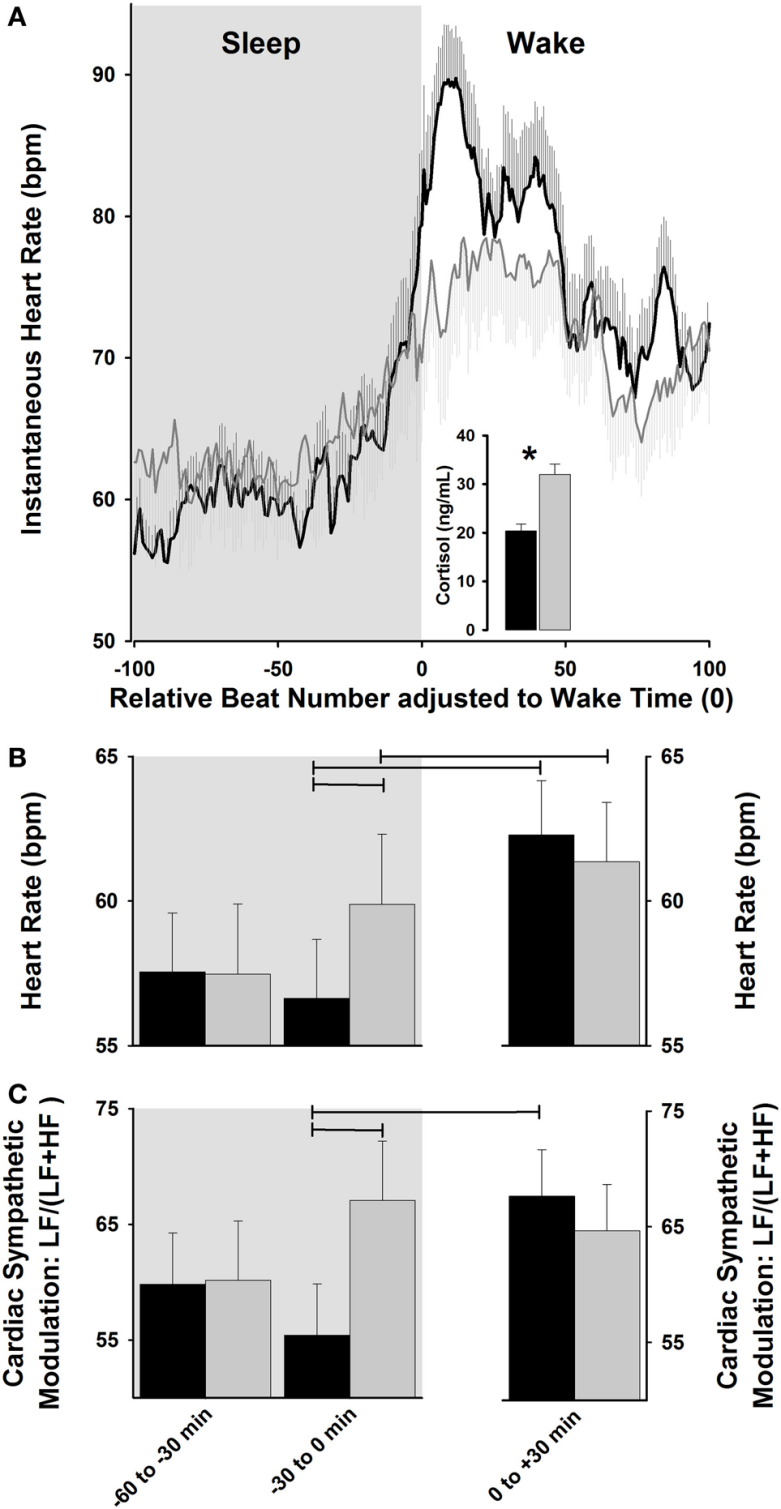
Cardiac modulation during sleep–wake transition. **(A)** Exposure to dawn stimulating light [dawn simulation light (DSL); gray lines] increases instantaneous heart rate (HR) relative to control dim light (black lines), and is associated with a higher cortisol increase. **(B,C)** DSL exposure (gray bars) progressively increases HR and relative cardiac sympathetic [LF/(LF + HF) ratio] levels during sleep-to-wakefulness, relative to control dim light (black bars). Horizontal lines: *p* < 0.05. Reproduced with permission from Viola et al. (82).

Collectively, light exposure impacts cardiovascular physiology, as indexed by its effects on HR and HRV, which may be associated with its effects on the underlying temporal orchestration set up by the endogenous circadian system (41, 70). In this context, strategies for the “optimizing” internal biological rhythms for regulation of cardiovascular events should lead to counteracting potential adverse events during the vulnerable morning hours. Interestingly, sleep–wake transitions in the early morning hours are associated with a relative increase in sympathetic activation in comparison to the rest of the day (81), which highlights the propensity for cardiac vulnerability upon awakening (66). Given the recent evidence for the progressive dynamics of light exposure on some surrogates of human peripheral physiology (e.g., cortisol levels) (53, 54), one may hypothesize that exposure to a dynamic rather than abrupt light exposure during the morning sleep–wake transition might impact on cardiovascular function, with a possible gradual rise HR and cardiac sympatho-vagal control. Indeed, one human study tested how HR and HRV was differentially impacted by exposure to a “naturalistic” dawn simulation light (DSL) source (progressive rise from 0 to 250 lux) with onset at 30-min before and offset at 30-min after scheduled wake-up time, as compared to a control dim light condition (82). Importantly, posture was controlled (recumbent during sleep and semi-recumbent during wake) and sleep duration was the same prior to wake-up time in both light conditions. Accordingly, DSL exposure gradually increased HR, as compared to a steeper HR increase under a control condition (82). These gradual light changes on HR dynamics were mirrored by gradual increases in cardiac sympatho-vagal modulation in DSL as compared to a control condition (Figure 2). While HRV is a potent tool typically used in physiological and pathological conditions (83, 84), it does not disentangle the interplay between sympathetic and parasympathetic cardiac control. These specific autonomic subsystems are modulatory reacting systems that control HR with different latent periods and time courses, such that parasympathetic effects on HR are much slower faster than parasympathetic effects (85). By applying a non-linear symbolic analysis method (86, 87), the relative sympathetic cardiac predominance was shown to be relatively stable between both experimental light settings, whereas the relative parasympathetic cardiac activity was stable only for DSL (82). Therefore, exposure to dynamic morning light simulating natural dawn might exhibit a protective effect on the heart by an evolving preparation of cardiac physiology for the wake-up process, which is likely achieved by a subtle pre-stimulation of cardiac activity during sleep prior awakening.

Despite the impact of light exposure on markers of cardiovascular control and the potential role for timed light exposure as a countermeasure against cardiac vulnerability in the morning hours, much remains to be established. Future studies in healthy normal and pathological aging, and in patients with increased cardiovascular risk (e.g., hypertensive patients and those with increased myocardial risk), may help to establish light as a countermeasure against the risk for acute morning cardiovascular events.

## Author Contributions

All authors listed have made a substantial, direct, and intellectual contribution to the work and approved it for publication.

## Conflict of Interest Statement

The authors declare that the research was conducted in the absence of any commercial or financial relationships that could be construed as a potential conflict of interest.
